# Reaction-diffusion hydrogels from urease enzyme particles for patterned coatings

**DOI:** 10.1038/s42004-021-00538-7

**Published:** 2021-06-29

**Authors:** Anthony Q. Mai, Tamás Bánsági, Annette F. Taylor, John A. Pojman

**Affiliations:** 1 Department of Chemistry & The Macromolecular Studies Group, Louisiana State University, Baton Rouge, LA USA; 2 Chemical and Biological Engineering, University of Sheffield, Sheffield, UK; 3 Department of Chemistry, University of Birmingham, Birmingham, UK

**Keywords:** Supramolecular chemistry, Gels and hydrogels

## Abstract

The reaction and diffusion of small molecules is used to initiate the formation of protective polymeric layers, or biofilms, that attach cells to surfaces. Here, inspired by biofilm formation, we present a general method for the growth of hydrogels from urease enzyme-particles by combining production of ammonia with a pH-regulated polymerization reaction in solution. We show through experiments and simulations how the propagating basic front and thiol-acrylate polymerization were continuously maintained by the localized urease reaction in the presence of urea, resulting in hydrogel layers around the enzyme particles at surfaces, interfaces or in motion. The hydrogels adhere the enzyme-particles to surfaces and have a tunable growth rate of the order of 10 µm min^−1^ that depends on the size and spatial distribution of particles. This approach can be exploited to create enzyme-hydrogels or chemically patterned coatings for applications in biocatalytic flow reactors.

## Abstract (web summary)

Enzyme reaction networks can control the spatial and temporal formation of hydrogels. Here, inspired by biofilm formation, urease-containing particles create a propagating pH gradient which induces thiol-acrylate polymerization, with properties tuned by solution viscosity, particle dimensions, and spatial configuration.

## Introduction

The immobilization of biomolecules such as enzymes in polymers and hydrogels is important for numerous applications including sensing and synthesis in biocatalytic flow reactors^1,2^. In natural systems, the immobilization of cells in hydrogels is often regulated by reaction and diffusion of small molecules in the extracellular solution. Microorganisms such as bacteria produce a sticky layer of extracellular polymeric substances that help them attach to surfaces in communities known as biofilms. Bacteria use an autocatalytic signaling messenger, or autoinducer, to initiate biofilm formation above a threshold group size in the phenomenon known as quorum sensing^3,4^. The biofilm protects the organisms against disturbances such as flow or harsh chemicals and ensures sustained bioactivity for continuous chemical processing in bacterial flow reactors^5^.

Methods for the production of structured enzyme-hydrogels, such as photolithographic masks or droplet microfluidics^6,7^, typically exploit UV and initiators or multiphase emulsion systems, thus immobilization of enzymes usually requires careful consideration of the conditions to prevent leaching and ensure degradation of the biomolecule does not occur^8^. Spatial control of the formation of hydrogels under physiological conditions would be particularly useful for coating enzymes-particles or surfaces and for applications in flow reactors, allowing for optimization of the biocatalytic process^9,10^. To this end, bioinspired processes combining reaction and diffusion are of increasing interest^11–14^.

In diffusion-control to form structured hydrogels, reactive components are spatially separated by initial localization of reactants^15^. Diffusion of species results in reaction and gelation that can be used to produce multilayer hydrogel systems or free-standing 3D supramolecular structures^16,17^. Acid diffusion provides a convenient means for triggering supramolecular self-assembly and changes of pH in space and time can be used to control the final hydrogel properties, including stiffness^18–21^. These systems typically rely on initially imposed gradients and might be distinguished from autocatalytic reaction-diffusion processes which involve the sustained generation of concentration gradients and chemical patterns by internal mechanisms^22–24^. On the other hand, enzyme-assisted self-assembly uses enzymes such as phosphatase to catalyze the formation of molecules that self-assemble in-situ and form a hydrogel^25,26^. The inactive precursors for gelation are contained in the solution or in a seed layer and the resultant hydrogels form layers that are nm to µm in size, primarily controlled by reaction and diffusion of the hydrogelators^27,28^.

Here, inspired by bacterial biofilms, we used enzyme particles to produce a propagating basic front that triggers the growth of hydrogel around the particles. This growth rate, 10 µm/min, is relatively fast and extends over cm distances as it relies on the reaction and diffusion of small molecules in the aqueous solution, rather than diffusion of hydrogelators. Urease, which catalyzes formation of ammonia from urea, was previously dissolved in solution and coupled with polymerization of a water-soluble thiol and polyethylene glycol diacrylate to form uniform hydrogels throughout the sample^29,30^. Now we show that the reaction is capable of generating sustained local polymerization fronts around an enzyme-loaded particle and binds the particles to surfaces.

The enzyme urease is prevalent in microorganisms and plants^31^. It is used by bacteria for protection against the acidic environment of the stomach and is implicated in the formation of mineralized biofilms^32^. The increase in pH combined with the typical bell-shaped rate pH curve of the reaction results in autocatalytic production of base and computer simulations have shown that cooperative effects are possible above a critical number or density of particles^33,34^. It has also been exploited in numerous material applications utilizing the change in pH in aqueous solutions at ambient temperatures^35–40^. These studies aid in the design of complex adaptive responses in soft matter systems inspired by nature^41^. Here, we combined simulations with experiments to determine the role of the autocatalytic reaction in the growth of the hydrogel, and how it depended on enzyme particle size.

One major drawback to the use of purified urease is that it tends to undergo degradation in solution and numerous immobilization supports and additives have been proposed that typically improve the longevity at the expense of the activity^42,43^. In order to circumvent these issues here, we used ground watermelon seeds as urease source^44^, which maintained the enzyme in its native environment in the particles. In addition, the seeds contained multiple enzymes creating the possibility of exploiting synergetic enzymatic processes in biocatalytic flow reactors for sensing or synthesis applications.

## Results

### Reaction-diffusion hydrogels from urease in ground watermelon seeds (WMS)

**Fig. 1:**
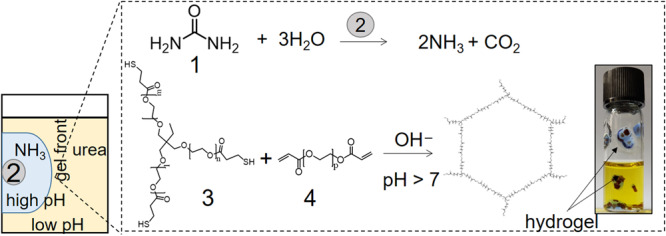
Scheme for the growth of reaction-diffusion hydrogels from urease enzyme particles. The two coupled reactions used were: 1 = urea, 2 = ground watermelon seed (WMS) containing enzyme urease, 3 = THIOCURE^®^ ETTMP 1300 and acid impurity (mercaptopropionic acid), 4 = polyethylene glycol diacrylate (PEGDA) 700. Image of vial (i.d. 10 mm) containing components for the reaction and pH indicator (bromothymol blue, pKa = 7.1) and hydrogels (blue) around the seeds after 30 min. The initial concentrations were: [urea] = 0.075 M, [PEGDA] = 0.2 M and [ETTMP] = 0.15 M and initial pH = 3.

The process for growth of hydrogels from enzyme particles is illustrated in Fig. 1. The aqueous solution containing urea, THIOCURE^®^ ethoxylated tri-thiol (ETTMP) 1300, and polyethylene glycol diacrylate (PEGDA) 700 was added to the urease-particles. The pH of the solution was initially low (~3) as a result of acid impurity in the ETTMP^29^. Ammonia was produced as a result of the urease-catalyzed hydrolysis of urea and raised the pH of the solution around the particle. Diffusion of base into the solution catalyzed the polymerization reaction, resulting in a traveling pH reaction front coupled to a polymerization front and a hydrogel layer formed around the particle.

In initial experiments, watermelon seeds were ground into mm-sized particles and placed in glass vials containing a solution of urea, ETTMP, PEGDA, and pH indicator. The vials of solution were placed on their side and enzyme particles were distributed evenly along the glass surface; when the vials were placed upright after 30 min, the particles surrounded by blue hydrogel remained attached to the surface in solutions containing all three components (Fig. 1). The thickness of the hydrogel around the particle depended on the reaction time and could be halted by removing the solution, revealing a dome-shaped hydrogel around the active ground seed.

**Fig. 2:**
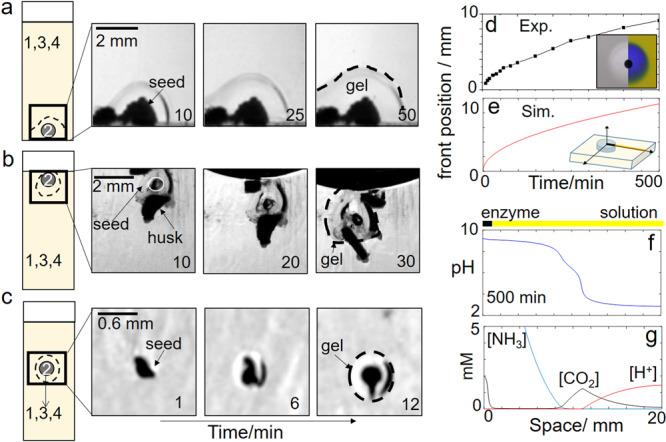
Growth of hydrogels from enzyme particles on surfaces, at the air–water interface or in motion. **a** Hydrogel on ground watermelon seeds (2) in a solution of 1 = urea, 3 = ETTMP 1300 (+ acid), 4 = PEGDA 700 with the initial concentrations: [urea] = 0.075 M, [PEGDA] = 0.2 M and [ETTMP] = 0.15 M. **b** Hydrogel on seeds trapped at the air-water interface along with inactive husk. **c** Hydrogel growth on a seed sinking to the bottom of the cuvette. **d** Position of hydrogel and pH front in time, inset shows solution with and without pH indicator (image size = 14 × 12 mm). **e**–**g** Reaction-diffusion simulations of the urea-urease reaction with particle diameter = 1 mm and with [urea] = 0.075 M, [H^+^] = 2 mM: **e** Position of pH front in time (pH > 7) and **f** pH profile and **g** ammonia, carbon dioxide, and acid profile at 500 min of reaction time.

The growth of the hydrogel around the ground watermelon seeds (WMS) is shown in a series of images in Fig. 2^29^. The hydrogels formed a layer around particles at the base of the cuvette (Fig. 1a), trapped at the air-water interface (Fig. 2b) or falling through the solution to the bottom of the cuvette (Fig. 2c). The position of the polymerization front was tracked in a series of images with and without indicator and plotted as a function of time (Fig. 2d). The pH front (blue) and the polymerization front (clear) propagated outward from the particles with the same speeds, hence showing that they were directly coupled, and the hydrogel fronts were indeed driven by the production of base from the urease-catalyzed reaction. Average velocities were of the order of 0.015 mm min^−1^ from 25 to 500 min.

The urease reaction is autocatalytic in base and in thin layers of urea-urease solution in a petri dish, basic reaction fronts propagate with constant velocities of 0.1–1 mm min^−1^ ^45^. The gelation fronts here should be distinguished from the autocatalytic reaction fronts as the catalyst is located within the enzyme particle thus the autocatalytic process cannot occur in bulk solution, only in the particle. The front velocity is also affected by the viscosity of the medium, which increased as a result of the macromolecules ETTMP and PEGDA, so the diffusion constant of base is lower than in aqueous solution.

### Comparison of reaction-diffusion pH fronts from urease-particles with diffusion of base alone

The front speed and profiles of species in the solution around the enzyme particle were determined using an eight variable reaction-diffusion model of the urease reaction developed in earlier work (see “Methods”):^45,46^

1 $$\frac{{\partial C}}{{\partial t}} = f(C) + D\left[ {\frac{1}{r}\frac{\partial }{{\partial r}}\left( {r\frac{{\partial C}}{{\partial r}}} \right)} \right]$$

where *C* is the concentration of species, *f*(*C*) contains enzyme reaction and equilibria terms, *D* is the diffusion coefficient, and space is given in radial coordinates, *r*, assuming cylindrical symmetry. In these simulations, enzyme was located at one end of the reaction domain with the urea and acid in the surrounding solution and diffusion coefficients were of the order of 1 × 10^−3^ mm^2^ min^−1^, taking an approximate value for diffusion of small molecules in the solution of macromolecules or hydrogel^47^. The simulations are not expected to quantitatively reproduce experiments, but allow us to determine expected trends in behavior with changing the enzyme-reaction conditions. Nevertheless, the front traveled with approximately constant velocity of 0.015 mm min^−1^ from 25 to 500 min in good agreement with the experiments. The position of pH front—where the pH increased above 7—is shown in Fig. 2e as a function of time.

The pH profile is shown at 500 min in Fig. 2f. The pH had values 9.2 in the vicinity of the particle and dropped to the surrounding solution pH of 3 further along the domain. A point of inflection can be observed in the pH profile around 6.5. This occurs because of the carbon dioxide that is also produced as a result of the urease reaction. The increase in pH is driven by the net production of two ammonia molecules (acid consuming) compared to one carbon dioxide molecule (acid producing). The CO_2_ diffuses from the particle into the acidic solution and slows the advancing ammonia front (Fig. 2g).

**Fig. 3:**
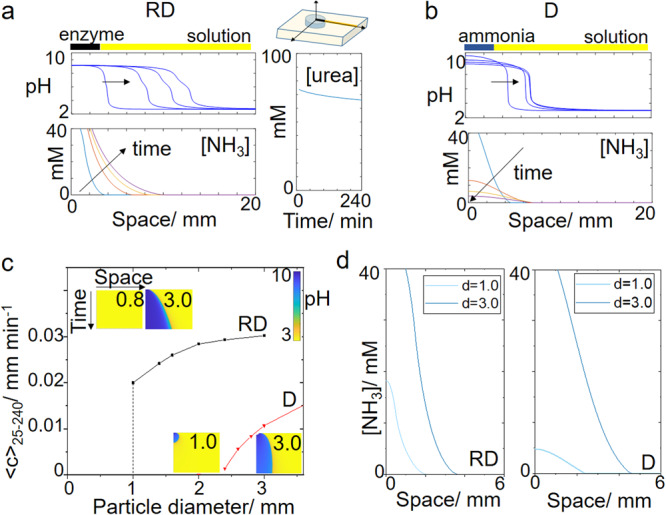
Simulations comparing reaction-diffusion (RD) of ammonia from an enzyme particle with diffusion (D) of ammonia alone. pH and ammonia profiles in space at 25, 95, 165, and 235 min from a particle of diameter *d* = 3.0 mm in **a** reaction with urease and **b** diffusion of ammonia. Also shown in **a** average concentration of urea in time in the solution. **c** Average front speeds (25–240 min) as a function of particle diameter. Insets show pH space-time plots for 20 mm × 240 min with particle diameter indicated. Color bar shows pH values. **d** Ammonia profile in space at *T* = 25 min for two different particle lengths. The initial concentrations were for RD: [urea] = 0.075 M and acid = 2 mM in solution and *E* = 100 unit g^−1^ in particle and D: [NH_3_] = 0.15 M in the particle and [H^+^] = 2 mM in solution.

We can use the simulations to compare the pH front from the enzyme reaction to the diffusion of ammonia alone. With reaction-diffusion from the catalytic particle, ammonia is continuously produced, leading to a propagating pH profile of approximately constant amplitude (Fig. 3a). The average concentration of urea in solution dropped to 83% of its original concentration in 240 min (Fig. 2g). The pH and polymerization fronts will continue to propagate until all the urea is consumed; indefinitely if urea is continuously supplied. In the case of diffusion alone, a particle was used containing 0.15 M ammonia, and the initial pH was 10.9. The ammonia rapidly decreased in time, and there was a reduction in amplitude of the pH profile until all the ammonia was depleted (Fig. 3b).

In both cases, the average speed of the front between 25 and 240 min increased with increasing particle diameter (Fig. 3c). The concentration profile of a species in space determines its rate of change as a result of diffusion (Eq. 1) and the maximum curvature of the profile increased as the particle diameter was increased (Fig. 3d). With reaction-diffusion, the front was only observed above a critical diameter of 1.0 mm. This is typical of an autocatalytic process in a particle where the removal of autocatalyst competes with its production resulting in an on–off, switch-like response^46^. In the urease reaction, which has a maximum rate at pH 7, the influx of acid from the solution inhibits the autocatalytic reaction in small particles <1.0 mm. With diffusion alone, there was simply insufficient base to create a propagating front from particles <2.4 mm (space–time plot in Fig. 3c).

These simulations demonstrated that reaction and diffusion of ammonia are more effective than diffusion alone at maintaining a constant amplitude pH profile for gelation from small enzyme particles over long timescales. Earlier simulations have shown that a cooperative effect is expected as a result of the local amplification and diffusion of ammonia in urease-particles, resulting in reaction-diffusion fronts or waves above a critical number or density of particles, and synchronizing activity across a heterogeneous population^34,48^.

### Control of hydrogel formation in arrays of urease particles

**Fig. 4:**
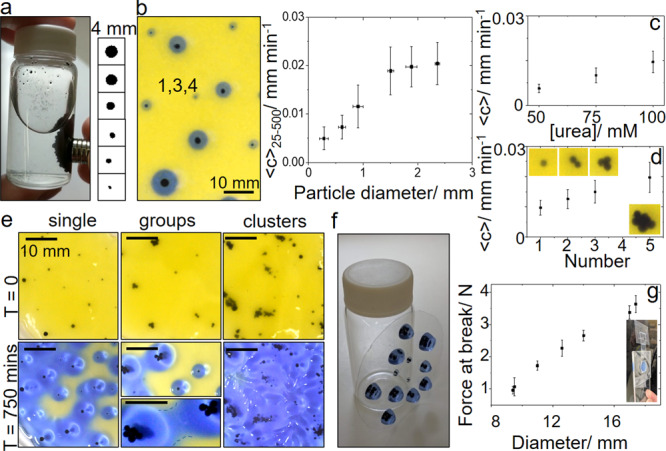
Patterned enzyme-hydrogels from arrays of urease-particles. **a** Magnetic urease-particles prepared from watermelon seed powder (WMSP) and iron oxide in agar and images of particles of size 0.1–2.5 mm. **b** Image of single magnetic enzyme particles placed in solution in a Petri dish with [urea] = 0.075 M, [ETTMP] = 0.05 M, [PEGDA] = 0.075 M and pH indicator and average velocity (25–500 min) of fronts as a function of particle diameter. Dependence of average velocities of the pH front on **c** the [urea] and **d** number of 1 mm particles placed in a group. **e** Particles arranged in different spatial configurations and hydrogel surface coverage at T = 750 min. Hydrogel bridge formation indicated in groups at *T* = 750 min. **f** Hydrogels remained attached to surface upon removal of solution. **g** Lap-shear test and tensile force at break as a function of hydrogel disk diameter. Scale bars on images correspond to 10 mm and error bars are standard deviations from three measurements.

To investigate the formation of hydrogels in spatial arrays of particles, magnetic particles were prepared from the ground watermelon seed (see “Methods”). The active powder was separated from the inactive husks and was mixed with iron oxide filings and agar, to create 100 µm to 3 mm-sized magnetic enzyme-containing particles (Fig. 4a). These particles were placed in a Petri dish with a thin layer of solution (2 cm) containing urea, ETTMP, PEGDA, and pH indicator (Fig. 4b). The average speed of the pH and hydrogel front increased from 0.005 to 0.02 mm min^−1^ with increasing particle diameter; similar values of the front speed were obtained in simulations. However, in contrast to the simulations, fronts were observed propagating from all particles including the smallest diameter used of 100 µm. This may arise as a result of changes in the diffusion properties of species in the enzyme particles, which were not accounted for in the simulations.

The rate of reaction and hence speed of pH fronts in the urease reaction can be controlled by the initial concentrations of acid, enzyme, and urea. The increase in front velocity with the concentration of urea is shown in Fig. 4c^33^. The speed of the pH fronts also increased with increasing numbers of particles in a group, similar to changing the size of a single particle (Fig. 4d). Alternatively, increasing temperature could be used to increase the propagation velocity however this may also lead to polymerization of the thiol-acrylate by a free radical mechanism in the bulk solution^49^.

The process can be used to obtained patterned enzyme-hydrogels on surfaces from an initial spatial configuration of the magnetic enzyme particles. Single particles placed with approximately uniform separation in a Petri dish gave a greater surface coverage of hydrogel at 750 min than a similar number placed in groups (Fig. 4e, single). Hydrogel bridges formed between individual particles or groups that accelerated the process (Fig. 4e, groups) and clusters of particles gave complete coverage of the surface with hydrogel at 750 min (Fig. 4e, clusters). The reaction could be stopped at any point by removal of the solution resulting in enzyme particle hydrogels that were attached to the surface where gelation took place (Fig. 4f). Lap shear tests were performed with gel disks of various sizes attached to strips of polyethylene (see “Materials and methods”) and failure loads are shown in Fig. 4h with strengths of the order of N cm^−2^.

### Chemically patterned enzyme hydrogels in flow reactors

**Fig. 5:**
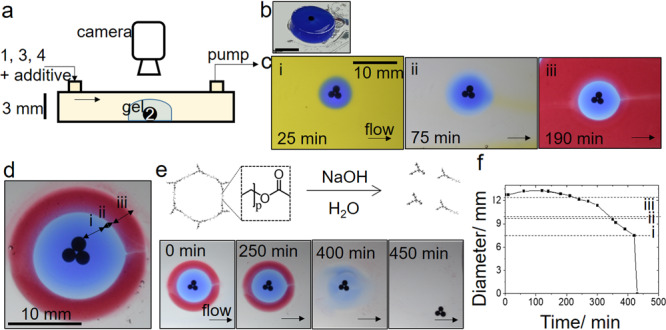
Chemically patterned enzyme hydrogels in flow reactors. **a** Set-up with solution pumped through containing [urea] = 0.12 M, [ETTMP] = 0.05 M, and [PEGDA] = 0.075 M and chemical additives. **b** Gel-disk formed in the reactor following removal of solution **c** Images to show chemical patterning of gel through addition of solution containing (i) pH indicator (ii) no indicator (iii) water-soluble oil paint and **d** Layered gel with (i) pH indicator, (ii) no additive and (iii) water-soluble oil paint. **e** Degradation of layered gel by base-catalyzed ester hydrolysis and images to show removal of layers. **f** Plot of the layered hydrogel diameter in time with base-catalyzed degradation in the flow reactor. Scale bars on images correspond to 10 mm.

We exploited the reaction-diffusion polymerization to obtain chemical patterning of the hydrogel around the enzyme particles in flow reactors (Fig. 5a). The magnetic enzyme particles were initially positioned using a magnet and upon addition of solution of urea, ETTMP, and PEGDA a hydrogel layer quickly formed adhering the particles to the surface. The polymerization front propagated out from the particles resulting in the formation of uniform hydrogel disks, with equal growth rates in all directions despite the flow (Fig. 5b).

For patterning of the hydrogel itself, a component could be easily incorporated into the gel by its addition to the inflow solutions. In Fig. 5c, we illustrate the principle with addition of pH indicator to the inflow solutions up to 25 min to three enzyme particles in a group. This was followed by addition of solution with no indicator resulting in a clear band (with some diffusion of indicator blurring the interface), then finally addition of a water-soluble oil paint resulting in a red band. The final patterned gel is shown in Fig. 5d with a symmetric structure around the enzyme particles.

The hydrogel and chemical additives could be removed from the reactor through base-catalyzed hydrolysis following addition of NaOH (pH = 14) to the flow (Fig. 5e). The ester linkages in both ETTMP and PEGDA undergo hydrolysis, and the lifetime of the gel can be tuned by the initial composition, in particular the ratio of ETTMP/urea, with lifetimes of hours to years obtained in the previous work^29^. The process is accelerated by addition of base. With inflow of NaOH here, first some swelling of the gel was observed, followed by slow degradation and then more rapid disintegration (Fig. 5f). This is typical of polymer degradation by bulk erosion, with the acceleration possibly attributable to percolation effects whereby macromolecular degradation products remain trapped in the hydrogel until a critical porosity is obtained^50^. Thus, in the flow reactor, first the chemical additives were liberated from the layers followed by the removal of the enzyme particles from the reactor. The enzyme particles could then be re-used in multiple experiments.

## Conclusions

We have demonstrated here how the urease enzyme might be exploited to produce hydrogel coatings on particles. The process was controlled primarily by the reaction and diffusion of small molecules, urea and ammonia, from urease enzyme particles and hydrogel growth rates, were of the order of 10 µm/min. This general method can be applied to any enzyme-producing acid or base coupled with pH-regulated gelation. Different enzymes could be spatially positioned in particles in the hydrogel layers, allowing for sequential control of the enzyme catalysis. The hydrogels can also be chemically patterned by addition of various components to the external solution.

The use of biocatalysts in flow reactors for sensing or synthesis applications frequently requires immobilization of purified enzymes in hydrogels which can result in loss of activity or leaching. Ground watermelon seeds were used for the production of enzyme particles, resulting in a stable and reusable source of the enzyme urease. In addition, the solid extracts obtained from watermelon seeds contain multiple enzymes and thus can be used in biocatalytic flow reactors where the natural synergic effects might be exploited for the synthesis of chemicals.

We were inspired by bacteria that form protective biofilms that bind the bacteria together at surfaces and interfaces. More complex responses are possible in systems with enzyme particles when the autocatalytic properties of the reaction can be harnessed^34^. This includes the switches, oscillations, and spatial waves that have been used to drive self-assembly and chemo-mechanical changes^24,51–55^. There are numerous other enzyme-catalyzed reactions that display autocatalysis and propagating reaction-diffusion fronts^56,57^. Then, similar to bacteria, the autocatalytic molecules may be used for signal processing and spatiotemporal programming of enzyme-hydrogels for applications in biotechnology and soft robotics^58^.

## Methods

### Materials

For the batch vial trials: WMS “Sugar Baby” containing the enzyme urease were obtained from Premier seeds direct (Wiltshire UK, 80 seeds/pack). For the magnetic particles and flow experiments: watermelon seeds “Crimson Sweet” were obtained from Eden Brothers (Arden, USA, I lB/pack). The water-soluble thiol-ethoxylated trimethylolpropane tri(3-mercaptopropionate) (THIOCURE^®^ ETTMP 1300, Mn (approx.) = 1300 g/mol, *ρ* = 1.15 g/ml)) was acquired from Bruno Bock Chemicals, and poly(ethylene glycol) diacrylate (PEGDA, Mn = 700 g/mol, *ρ* = 1.12 g/ml) was purchased from Sigma Aldrich and contained MEHQ and BHT inhibitor. THIOCURE^®^ ETTMP contains <1% 3-mercaptopropionic acid 3-MPA (pKa = 4.34) as impurity and the pH of the sample was determined before experiments. The average pH of three solutions of [ETTMP] = 0.15 M in water was found to be: 2.73 ± 0.02. Extra pure (>98%) urea and the indicator bromothymol blue pH indicator (pKa 7.1) or bromocresol purple (pKa 6.3) were purchased from ACROS Organics. Black iron oxide powder was purchased from Alpha Chemicals (Missouri, USA) and agar powder was obtained from Living Jin (San Jose, USA). The xantham gum was obtained from Now^®^ Foods. The Duo-aqua oil water-soluble oil paint was obtained from HK Holbein (USA). Ultrapure water (18.2 MΩ cm) was utilized in experiments and all chemicals were used as purchased without further purification.

### Hydrogel formation from ground watermelon (WMS) seeds

Five reaction solutions (A–E) were prepared using a 1. urea (200 µL, 2 M stock solution), 3. ETTMP (900 µL), 4. PEGDA (700 µL) to give a mixture of (A) 1 and 3; (B) 3; (C) 1,3 and 4, (D) 3 and 4, (E) 4 and the total volume of each solution was made up to 5.3 ml with ultrapure water. Bromothymol blue was added to the water as pH indicator (0.8 g L^−1^ or 0.08%) and the solution was mixed until transparent. The initial concentrations were (where present) [urea] = 0.075 M, [PEGDA] = 0.2 M, and [ETTMP] = 0.15 M. Watermelon seeds (Sugar Baby) were ground by hand for 5 min and glass vials were prepared to contain 0.1 g ground WMS to which the reaction solution (A–E) was added. The vials were placed on their side and the seeds spread along the bottom surface. After 1 h, the vials were placed upright and images were obtained of the result. The rate of gelation of ETTMP and PEGDA depends on pH and temperature and was triggered here by the increase of pH in vial C as a result of the urea-urease reaction. Experiments were performed at room temperature (20 ^o^C).

Shadowgraphy was used to image changes in refractive index with density, resulting in a dark band at the gel–solution interface. The position of the gel front could thus be tracked in the absence of indicator. Briefly, as described in our earlier work^29^, experiments were performed in a glass cuvette, containing ground WMS and reaction solution, placed in front of a 200 mm focal length plano-convex condenser lens (Edmund Optics). For illumination, a MiniSun A4 LED light pad and a white LED (diameter: 5 mm, epoxy dome removed) were used. A series of images was obtained using a PixeLink CCD camera connected to a computer and processed using ImageJ. In these experiments, we also observed moving dark bands in the bulk solution arising as a result of convection in the reaction mixture, possibly driven by evaporation of solution. This could be reduced through the addition of xanthan gum.

### Preparation of magnetic WMSP-agar particles

The watermelon seeds (Crimson Sweet) were milled for several minutes and then acetone was added. When immersed in the solvent, the seed husks settled and a tan particulate phase stayed dispersed in the acetone. Buchner filtration of the supernatant and air drying overnight yielded a fine watermelon seed powder (WMSP) about 25–30% by weight of the starting seeds with particles of size 1–10 µm (Supplementary Fig. 1). Using Nessler’s reagent, assays of the urease enzyme contained in these particles produced around 170 mg of NH_3_ per g of WMSP in 5 min and thus an activity of the order of 2000 units g^−1^ compared to pure urease activity of 600,000 units g^−1^. There was no significant difference between the activity of Crimson Sweet and several other varieties of watermelon seed that were tested (Supplementary Table 1). The remaining husks were also analyzed and showed little to no catalysis of urea hydrolysis. The dry powder was stored at room temperature for 350 days without significant degradation (Supplementary Table 2).

The magnetic agar-WMSP particles were prepared, as detailed in the Supplementary information, using 66 g agar solution (with 2.5 agar); 20 g WMSP suspension (with 5 g WMSP) and 14 g iron solution (with 4 g Fe_3_O_4_) at 55 °C. The mixture was added to vegetable oil with blending at 500 rpm and the resultant particles were in the 100 µm to 5 mm diameter range. The particles were cooled in an ice bath to 10 °C and then washed with 200 mL hexane and stored in hexane for use. Hence the WMSP content was typically 5 g of 2000 u g^−1^ in 100 g particles so 5% w/w WMSP or 100 unit g^−1^ particle, as prepared. A comparison of the activity of WMSP, magnetic WMSP-agar particles, and purified urease in unbuffered urea solutions is shown in Supplementary Fig. 2. In experiments, the magnetic enzyme particles were placed in Petri dishes of diameter 8 cm and a solution containing [urea] = 0.05 M, 0.075 or 0.1 M, [ETTMP] = 0.05 M and [PEGDA] = 0.075 M, bromocresol purple indicator (1% in ethanol), 1% antifoam and xantham gum was added to give layer depth of 2 cm. Solutions were degassed before use and particles were positioned individually, in groups or in clusters. Images of the experiments were obtained every minute and ImageJ was used for the determination of front speed and particle size. Error bars correspond to standard deviations from measurements with at least three particles or groups of particles.

### Mechanical testing

Lap shear tensile tests (based on ASTM F2255-03) were performed of various sized thiol-acrylate hydrogel disks inset into plexiglass. A solution was prepared containing urea (1.8 g), ETTMP (13.2 g), PEGDA (10.57 g), 180 g H_2_O (Nanopure^TM^), three drops 1% antifoam AF, xanthan gum (0.51 g), and 5–10 drops of 1% bromocresol purple, give final volume of 201 mL and concentrations of [urea] = 0.15 M, [PEGDA] = 0.075 M and [ETTMP] = 0.05 M. The monomer solution was pipetted in disks of varying diameters created from 1/8” plexiglass. The mixture was spiked with 10% NH_4_OH(aq) solution at a ratio of 50 µL to 1 g of solution. A thin strip of polyethylene of widths corresponding to the hydrogel diameter was placed on top of the disc solution, and the reaction mixture was allowed to cure. By pulling on the flexible polyethylene, adhesive strength was determined via a single-lap shear test on an Instron 5582 equipped with a 2 kN load cell at a constant ramping rate of 2 mm/min for the various overlapped areas. Error bars correspond to standard deviations from measurements from experiments performed in triplicate.

### Formation of hydrogel in a flow cell

A rectangular flow cell was constructed with two ports (in and out) that take a 1/4 inch OD PVC tubing, connected to a peristaltic pump. The top plate was made of 1/8 inch polycarbonate, with a 1/16 inch silicone gasket, and glass was used for the bottom/sides (1/8 inch thick). The gap width of the cell was 3 mm and the total volume of the cell was around 26 mL. The magnetic WMS-agar particles were added to the flow cell in a urea/ETTMP/PEGDA solution and positioned using a magnetic bar. This solution consisted of: 89.8 g H_2_O, 6.5 g ETTMP, 5.25 g PEGDA, 750 mg of urea, 0.25% xanthan gum to give a total volume 100 mL and final concentrations of [urea] = 0.12, [ETTMP] = 0.05 M and [PEGDA] = 0.075 M. The xanthan gum was included to increase the viscosity of the mixture. Solution was then pumped through at a rate of 125 mL min^−1^. Three different gel layers were formed around the particles, firstly bromocresol purple was used to visualize the pH change (solution was yellow, the gel was blue due to the formation of ammonia) and gel formation, a second layer of no indicator created a clear layer, and the third layer was red due to a water-soluble oil color. This final red colorant was chosen as to not have bleeding affects between the previous two layers. These layered particles then were subjected to a cycling flow of 0.1 N NaOH, which dissolved the layers and released the particles. We note that the gelled particles could not be removed even with flow rates up to 2 L min^−1^.

### Reaction-diffusion simulations

Numerical simulations were performed using MATLAB (solver ode15s) and an 8-variable model of the urea-urease reaction introduced in earlier work^45^. We assumed cylindrical symmetry and space was resolved along a radial profile with 215 × 0.1 mm cells; the first cells corresponded to the enzyme particle and the rest of the cells corresponded to the bulk solution (no enzyme). Qualitatively similar results were obtained with a 1D coordinate system for the enzyme particle or radial coordinates with spherical symmetry (Supplementary Fig. 3). The initial conditions in the solution were: [urea] = 0.075 M and [H^+^] = 2 × 10^−3^ M and in the particle: [urease] = 100 units/g and [H^+^] = 1 × 10^−7^ M. For diffusion of ammonia alone, the initial conditions in the particle were: [urease] = 0 and [NH_3_] = 0.03 M and in the solution: [H^+^] = 2 × 10^−3^ M. A central finite difference approximation was applied for space with the diffusion coefficients of species set at *D*_s_ = 1 × 10^−3^ mm^2^ s^−1^ and *D*_H+_ = 2*D*_s_. No-flux boundaries (*dC/dr* = 0) were applied at both ends of the 1D domain. For further details, see the Supplementary Information Model and Simulations.

## Supplementary information

Supplementary information

## Data Availability

The MATLAB code used in the current study is available upon reasonable request from the corresponding authors.

## Data availability

The datasets generated during and/or analyzed during the current study are available from the corresponding author on reasonable request.
