# Supplementary material for: Reaction-diffusion hydrogels from urease enzyme particles for patterned coatings
Source: Commun Chem. 2021 Jun 29;4:101. doi: 10.1038/s42004-021-00538-7 (PMC9814597; doi:10.1038/s42004-021-00538-7)
Supplement: Supplementary file 1 — Supplementary information [file 42004_2021_538_MOESM1_ESM.pdf]

Supplementary Information

Reaction-diffusion Hydrogels from Urease Enzyme-Particles for Patterned Coatings

Anthony Mai[1], Tamás Bánsági Jr.,[2,3] Annette F. Taylor[2]*, John A. Pojman Sr.[1]*

1 Department of Chemistry & The Macromolecular Studies Group, Louisiana State University, Baton Rouge, Louisiana 70803, USA

2 Chemical and Biological Engineering, University of Sheffield, Sheffield S1 3JD, UK

3 Department of Chemistry, University of Birmingham, Birmingham, UK

*A.F.Taylor@sheffield.ac.uk and john@pojman.com

Contents

Supplementary Methods 1

Production and analysis of urease enzyme-particles from watermelon seeds 1

1.1 Preparation of watermelon seed powder 1

1.2 Determination of the urease activity in watermelon seed power 2

1.3 Enzyme leaching from watermelon seed powder 3

1.4 Storage and stability of watermelon seed powder 3

1.5 Comparison of activity of purified urease, WMSP and magnetic WMSP-agar particles 4

Model and Simulations 5

Supplementary References 7

Supplementary Methods

Production and analysis of urease enzyme-particles from watermelon seeds

1.1 Preparation of watermelon seed powder

Watermelon seed kernels contain typically 35% protein and 50% lipids, along with fibre.[1] A batch of delipidated watermelon seed powder (WMSP), or seed meal, was prepared from 100 – 400 g of watermelon seeds purchased from Eden Brothers®.[2] The watermelon seeds were ground for up to two minutes using a flour mill at 25000 rpm, taking care to ensure the powder does not overheat. Acetone was added to the ground seeds using a ratio of 2 to 1 by volume acetone to ground watermelon seeds, and the mixture was left stirring overnight at room temperature. The husks were removed by passing the acetone mixture through a 120 mesh filter screen. The remaining turbid solution was then filtered through a Buchner funnel with a Whatman Grade 1: 11 µm filter paper. The filtrate was re-filtered through the wet-cake to recover all particles and the wet-cake was washed with

fresh acetone and then dried in air overnight. The yield of watermelon seed powder (WMSP) was 25% by weight of the initial seeds.

The resultant dry particles were examined using a Nikon Eclipse 50i microscope with 20X objective and a Nikon DS-Fi1 camera and were spherical in shape and of size 1 – 10 µm (Supplementary Figure 1). The mean diameter determined by a Coulter LS200 laser diffraction particle size analyzer was 4.8 µm. Similar sized particles were obtained by applying the same preparation method to different varieties of watermelon seed including Crimson Sweet, Sugar Baby, Jubilee, Tendersweet Orange and Black Diamond Yellow Belly. The particles obtained resemble the protein microbodies, or glyoxysomes, that are found in the cotyledons of watermelon seedlings and some other plants.[3] Typically they contain catalase and other enzymes involved in carbohydrate and fat metabolism.[4]

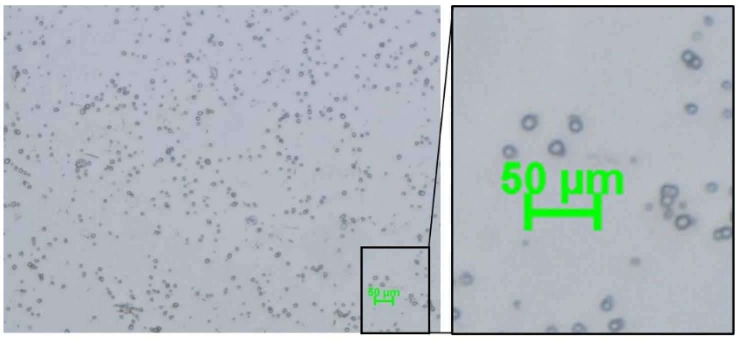

Supplementary Figure 1. Microscope image of the watermelon seed powder.

1.2 Determination of the urease activity in watermelon seed power

The catalytic activity of the enzyme urease was determined from the rate of production of ammonia from urea, where one unit of activity corresponds to 1 µmol (0.017 mg) of ammonia produced per minute under the specified reaction conditions – here pH 7 (phosphate buffer) and 25 ˚C. The mass of ammonia produced in 5 minutes was determined using Nessler's reagent and the absorbance was measured at 420 nm on a Vernier UV-Vis spectrophotometer.[5] A calibration curve was produced using ammonium sulfate as a standard. The WMSP was washed twice with 200 mL of water before use. The remaining husks from the preparation process were also analyzed and showed little to no activity. Assays were performed in triplicate with 10 mg of WMSP and the mean activity and standard deviation determined. The means were compared using a two-sample t-test with $p < 0.05$ as significant. The Crimson Sweet variety, which was used in the experiments described here, had activity of the order of 177 mg NH_3 per g WMSP in 5 minutes or 2080 units g^{-1} WMSP (Table S1). There was no significant difference ($P > 0.05$) between the activity of Crimson Sweet and the other varieties tested.

Supplementary Table 1. Average activity of urease in WMSP and comparison between some varieties of watermelon and standard deviation from assays performed in triplicate. The specific activity is expressed in units/g watermelon seed powder.

Variety	Rate (mg NH_3/ g WMSP/5 min)	Specific activity u g^{-1} WMSP
Crimson sweet	177 ± 20	2080 ± 233
Jubilee Improved	177 ± 17	2080 ± 204
Tendersweet orange	161 ± 2.6	1890 ± 31
Black Diamond Yellow Belly	179 ± 6.7	2097 ± 78

Three separate batches of Crimson Sweet were tested and there was no significant difference between the mean activities (172, 179 and 171 mg NH3 /g WMSP/5 min). Urease, a globulin, was

previously extracted and purified from watermelon seed meal using a salt or strong buffer, and the yield after purification was 0.253 mg from 50 g meal (Gaza 1).[6] The activity of the crude extract was 18000 units g^{-1} and once purified it was 150000 units g^{-1} protein, using a 3 minute assay at 37 ˚C. Pure crystalized urease from Jack Bean has activity of 600000 units g^{-1}, but urease powder type III from Jack Bean, as purchased from Sigma Aldrich, typically contains additional components from the purification procedure and has an average specific activity of 15000 - 50000 units g^{-1} with a 5 minute assay at 25 ˚C and phosphate buffer. Hence the WSMP used here has an activity around 4 - 13% of commercial Jack Bean urease type III.

1.3 Enzyme leaching from watermelon seed powder

The possibility of urease leaching from the WMSP into solution was investigated in several different batches of the prepared powder. Dry seed powder (0.5 g) was added to 30 g deionized water and stirred overnight. The solution was filtered through 0.45 µm syringe filters and 200 µL of this filtrate was assayed (section 1.2). Three batches of filtrate had less than 0.2% activity compared to the original WMSP, however one batch showed activity in the filtrate. It was ascertained that this batch of WMSP may have been damaged during the milling process. To determine if the leaching could be eliminated, 10 g of powder from this batch was soaked in water for 1 day, filtered, then treated with further two washes in 200 mL of acetone, and then 1 g was stirred in 15 g water for two hours and syringe filtered. The activity of the recovered WMSP was reduced by 10% compared to the original sample but there was no activity recorded in the filtrate in this case.

1.4 Storage and stability of watermelon seed powder

The recommended storage temperature of commercial Jack Bean urease powder type III (Sigma) is 2 – 8 ˚C, and of pure urease crystals is -20 ˚C. The activity of the WMSP was compared in dry samples stored in capped vials in the refrigerator (5 – 10 ˚C) and at room temperature for 350 days. We found that the activity was 100 ± 3% of the original activity (from assays performed in triplicate) after 350 days when stored in a refrigerator and 96 ± 3% when stored at room temperature. Hence the dry WMSP can be stored as used with little reduction in activity over at least this timescale.

Aqueous solutions of urease are known to degrade rapidly, even when stored in the refrigerator.[7] This can occur as a result of contamination with bacteria, or denaturing of the protein in solution over time. Samples of 0.5 g WMSP were placed in 15 g deionized water and stored in the refrigerator for around 30 days. One sample was treated with 0.5% phenoxyethanol, a germincidal glycol ether. We found that refrigerated aqueous suspensions maintained activity of 79 ± 2% after 28 days, and with addition of germicide, the activity was 88 ± 3% at 33 days. This is an improvement compared to solutions of extracted, purified urease in earlier work, however the half-life of urease in solution depends on the concentration and presence of additives.

Supplementary Table 2. Activity of dry or aqueous watermelon seed powder (WMSP) after storage, refrigerated or at room temperature (RT). Standard deviation was calculated from assays performed in triplicate.

Storage treatment	Relative activity/ %
Dry WMSP refrigerated, 350 days	100 ± 3
Dry WMSP RT, 350 days	96 ± 3
Aqueous WMSP refrigerated, 28 days	79 ± 2
Aqueous WMSP refrigerated + 0.5% germicide, 33 days	88 ± 3

1.5 Comparison of activity of purified urease, WMSP and magnetic WMSP-agar particles

An agar solution was prepared of 2.5 g agar in 63.5 g H_2O and heated to 91 ˚C for at least 30 minutes; then cooled to < 60 ˚C for WMSP/iron oxide addition. Two suspensions were prepared and vortex mixed for 30 s: 5 g WMSP in 15 g H2O at 55 ˚C (maintaining temperature to prevent gelation upon addition to agar) and 4 g Fe_3O_4 powder in 10 g H_2O at 55 ˚C. The WMSP mixture was added to agar mixture and homogenized at 5000 rpm for 10 s and then the Fe_3O_4 mixture was added to the agar/WMSP mixture. The mixture was dispersed at 1500 rpm for 10 s with a dispersion blade. Using vegetable oil as the outer phase, we created a suspension of the aqueous agar/WMSP/iron oxide in oil. Using a turbine blade at the bottom of a 500 mL beaker with 300 mL oil at 500 rpm yielded particles in the 100 micron to 5 mm diameter range. After the mixture was poured into the vegetable oil, we cooled the oil in an ice bath to 10 ˚C, still stirring. Once cooled, the agitation was stopped and most of the supernatant oil was decanted for later re-use. The remaining oil and particles then can be screen filtered and washed with hexane. We usually performed three 200 mL hexane washes of the particles. After the particles have been washed, they can then be stored in hexane for use and particles could be used multiple times. The WMSP content was typically 5 g of 2000 u/g in 100 g particles so 5% w/w WMSP or 100 unit/g particle, as prepared.

The standard assay gives a useful comparison of the activity under certain specified conditions, but it is not the same in experiments which are not buffered. We can compare the rate of production of ammonia through the change in pH in un-buffered solutions the agar particles, the WMSP and with purified enzyme (Jack Bean urease type IX, Sigma, with activity 76440 u/g solid). The solutions were prepared to obtain the same enzyme activity of 3.54 units/ml with the appropriate mass of WMSP or purified urease. The total volume of solution was 60 mL with 5.7 mM of urea and pH = 4 (adjusted using HCl). The solution was stirred with a magnetic stirrer at 300 rpm and pH monitored using a pH probe. For the same u/ml measured in the standard assay, and the concentrations used here, the WMSP-agar particle reaction results in a slightly slower increase in pH, compared to the WMSP and the profile is different in both cases compared to the free enzyme (Supplementary Figure 2).

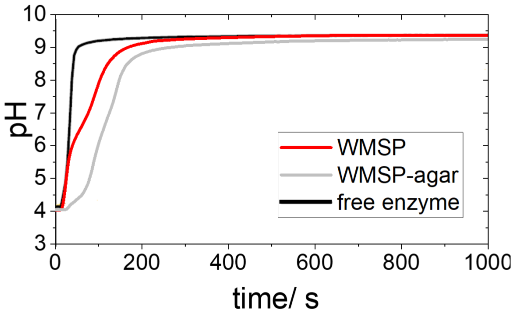

Supplementary Figure 2. Comparison of pH clock reaction in stirred, un-buffered solutions of 5.7 mM of urea, 3.54 u/ml urease and pH = 4 (HCl) with urease in agar-WMSP particles, WMPS alone and purified urease enzyme (Jack Bean urease Type III).

The rate of production of ammonia is affected by many parameters including the pH, the type of buffer, the ionic strength etc as well as enzyme immobilization and the mass transfer rates of solutes between particles and the urea solution, and thus stirring rate and surface area of particles. It is not possible to independently determine all of the enzyme kinetic parameters and role of mass transfer under the non-standard conditions employed in our experiments. However, a modified Michaelis-Menten expression was used in simulations in previous work and the maximum rate was optimized to give a reasonable fit to the experimental results.[8]

Model and Simulations

The purpose of the simulations was to compare the average velocity (over a given time period) of the reaction-diffusion front from the enzyme-particle with diffusion of ammonia alone from an ammonia-loaded particle. We also examined the pH profile, ammonia profile and urea profile in space in the simulations in order to establish how far the pH front may propagate before depletion of urea or ammonia occurs.

The urease reaction was modelled taking into account the following reactions:[8]

(SE1) $CO(NH_2)_2 + H_2O \xrightarrow{urease} 2NH_3 + CO_2$

(SE2) $NH_4^+ \rightleftharpoons NH_3 + H^+$ pK$_a$ = 9.25, rate2 = $k_2[NH_4^+] - k_{2r}[NH_3][H^+]$

(SE3) $CO_2 + H_2O \rightleftharpoons H^+ + HCO_3^-$ pKa = 6.35, rate3 = $k_3[CO_2] - k_{3r}[HCO_3^-][H^+]$

(SE4) $HCO_3^- \rightleftharpoons H^+ + CO_3^{2-}$ pKa = 10.25, rate4 = $k_4[HCO_3^-] - k_{4r}[CO_3^{2-}][H^+]$

(SE5) $H_2O \rightleftharpoons H^+ + OH^-$ pKa = 14, rate5 = $k_5 - k_{5r}[OH^-][H^+]$

where the enzyme rate is given by modified Michaelis-Menten kinetics:

$$v = \frac{k_1 E_T U}{\left(K_M + U(1+\frac{U}{K_S})\right)\left(1+\frac{[NH_4^+]}{K_P}\right)\left(1+\frac{K_{es2}}{[H^+]}+\frac{[H^+]}{K_{es1}}\right)} \quad \text{(SE6)}$$

and k_1 is the turnover number (s^{-1}), $[E]_T$ is the concentration of enzyme (M), K_M is the Michaelis constant, K_{es2} and K_{es1} are protonation equilibria of the substrate-enzyme complex. As we were interested in general trends here, we took the values of the enzyme constants from previous work: K_M = 0.003 M; K_{ES1} = 5 x 10^{-6} M; K_{ES2} = 2 x 10^{-9} M. For particles, the value of k_1 = 2.2 x 10^{-6} unit^{-1} g M s^{-1} was used with [E] recorded in u/g so that the product k_1[E] gives the maximum rate, v_{max} in M s^{-1}. The activity of urease in units is typically used in experiments rather than concentration when the enzyme source is not pure. We did not take into account substrate and product inhibition, as it plays a small role in the behavior, and we also omitted the transfer of NH_3 and CO_2 to the gas phase. The values of $k_2 - k_5$ are well established in the literature: k_{2r} = 4.3 x 10^{10} M^{-1} s^{-1}; k_2 = 24 s^{-1}; k_{3r} = 7.9 x 10^4 M^{-1} s^{-1}; k_3 = 0.037 s^{-1}; k_{4r} = 5 x 10^{10} M^{-1} s^{-1}; k_4 = 2.8 s^{-1}; k_{5r} = 1 x 10^{11} M^{-1} s^{-1}; k_5 = 1 x 10^{-3} s^{-1}.

Simulations of the resultant rate equations in three spatial dimensions over cm length scales are highly computationally intensive. However, given the inherent spatial symmetry in RD systems, some simplifications can be used. In previous work, in order to model the reaction in thin layers of solution in a petri-dish, we used the reaction-diffusion partial differential equation (PDE) equation in 1D:[8]

$$\text{(SE7)} \quad \frac{\partial C}{\partial t} = f(C) + D\frac{\partial^2 C}{\partial x^2}$$

where C is the concentration of species, f(C) is the reaction term, D is the diffusion coefficient and x is spatial coordinate. The model reproduced the experimentally observed trends of wave speed versus initial concentrations. For urease-loaded enzyme particles in solution, we used a three variable scaled model in radial coordinates with spherical symmetry.[9] The RD equation with cylindrical or spherical symmetry is given by:[10]

$$\text{(SE8)} \quad \frac{\partial C}{\partial t} = f(C) + D\left[\frac{1}{r^a}\frac{\partial}{\partial r}\left(r^a \frac{\partial C}{\partial r}\right)\right]$$

Where r is the radial coordinate and a = 1 for cylindrical and a = 2 for spherical symmetry. The 3 variable model with spherical symmetry was able to reproduce qualitative features of the experiments, and showed a change in the behavior with particle radius.

We compared the eight variable model of an enzyme particle in solution in 1D with radial coordinates and cylindrical or spherical symmetry. The equations were solved using MATLAB solver ode15s and a central finite difference approximation for space from grid points i = 1 .. N:

$$\text{(SE9)} \quad \frac{1}{r^a}\frac{\partial}{\partial r}\left(r^a \frac{\partial C}{\partial r}\right) = \frac{a}{r}\frac{dC}{dr} + \frac{\partial^2 C_i}{\partial r^2} = \frac{1}{ih^2}((i+\frac{a}{2})C_{i+1} - 2iC_i + (i-\frac{a}{2})C_{i-1})$$

where h is the spatial step size = 0.1 mm and the total length of domain is given by hN. The radius of enzyme-particle was hM where the grid points i = 1 .. M contained enzyme. The boundary conditions were no flux $dC/dr = 0$: $C_0 = C_2$ at i =1 and $C_{N+1} = C_N$ at i = N. The initial conditions in the solution were: [urea] = 0.075 M and $[H^+] = 2 \times 10^{-3}$ M and in the particle: [urease] = 100 units/g and $[H^+] = 1 \times 10^{-7}$ M. For diffusion of ammonia alone, the initial conditions in the particle were: [urease] = 0 and $[NH_3]$ = 0.15 M for radial and in the solution: $[H^+] = 2 \times 10^{-3}$ M. The diffusion coefficients were $D = 1 \times 10^{-3}$ mm^2 s^{-1}, except for H^+ which was 2×10^{-3} mm^2 s^{-1}. The position of the reaction-diffusion front was defined as the grid point where pH > 7 and the average front speed was determined from the change in front position over a specified time period.

For the autocatalytic reaction with enzyme dissolved in solution, the RD front propagated in 1D with constant profile and velocity (Supplementary Figure 3(a)). When the autocatalytic reaction was confined to an enzyme-particle in 1D (a slab) or radial coordinates with cylindrical or spherical symmetry, the pH profile became less sharp and the front slowed down as it propagated into the enzyme-free solution (Supplementary Figure 3(b – d). The results were qualitatively the same in all three cases, however the average front velocity from 25 – 500 minutes decreased as the coordinate system was changed from 1D to cylindrical and then spherical symmetry, reflecting the effective increase in the rate of diffusion. The velocity was an order of magnitude lower than for the autocatalytic pH fronts in solution.

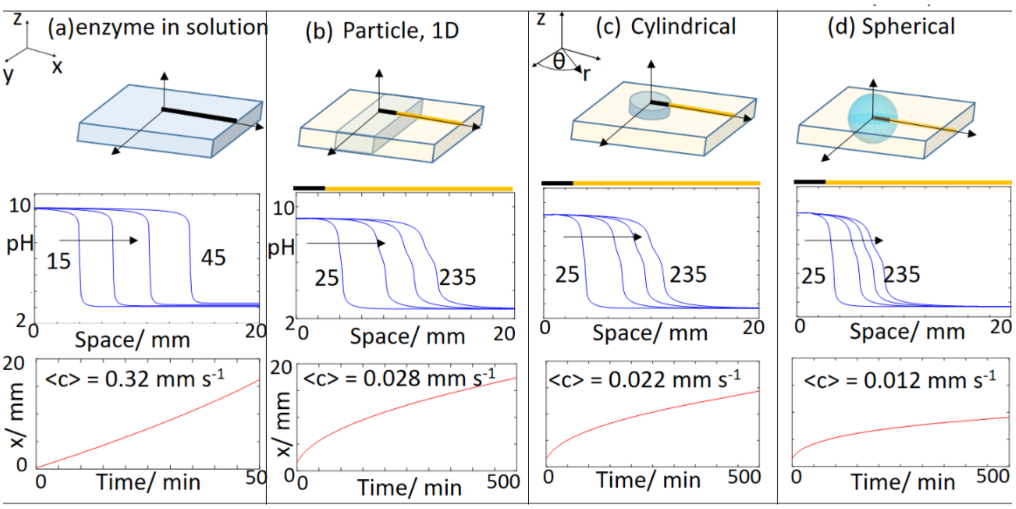

Supplementary Figure 3. Comparison of reaction-diffusion pH front propagation in simulations showing pH profiles in space (with time in minutes indicated) and position of pH fronts in time for (a) autocatalytic front in 1D with enzyme in solution, [E] = 4 u ml^{-1} (b) enzyme in particle, 1D (c) enzyme in particle with cylindrical symmetry (d) enzyme in particle with spherical symmetry. (b – d) Particle diameter = 3 mm and [E] = 100 u g^{-1}.

Supplementary References

1 El-Adawy, T. A. & Taha, K. M. Characteristics and composition of watermelon, pumpkin, and paprika seed oils and flours. *J. Agric. Food. Chem.* **49**, 1253-1259, doi:10.1021/jf001117+ (2001).

2 Mai, A. Q. Q. *Characterization, Immobilization, and Polymer Related Applications of Watermelon Seed Powder, a Practical Source of Urease Enzyme* PhD thesis, LSU, (2021).

3 Frederick, S. E., Gruber, P. J. & Newcomb, E. H. Plant microbodies. *Protoplasma* **84**, 1-29, doi:10.1007/BF02075940 (1975).

4 Kagawa, T., McGregor, D. I. & Beevers, H. Development of enzymes in the cotyledons of watermelon seedlings. *Plant Physiol.* **51**, 66-71 (1973).

5 Thompson, J. F. & Morrison, G. R. Determination of Organic Nitrogen. Control of Variables in the Use of Nessler's Reagent. *Anal. Chem.* **23**, 1153-1157, doi:10.1021/ac60056a029 (1951).

6 Mohamed, T. M., Mohamed, M. A., Mohamed, S. A. & Fahmy, A. S. Purification of urease from water melon seeds for clinical diagnostic kits. *Bioresour. Technol.* **68**, 215-223, doi:10.1016/S0960-8524(99)00157-1 (1999).

7 Bubanja, I. N., Bánsági, T. & Taylor, A. F. Kinetics of the urea–urease clock reaction with urease immobilized in hydrogel beads. *Reaction Kinetics, Mechanisms and Catalysis* **123**, 177-185, doi:10.1007/s11144-017-1296-6 (2018).

8 Wrobel, M. M. *et al.* PH wave-front propagation in the urea-urease reaction. *Biophys. J.* **103**, 610-615, doi:10.1016/j.bpj.2012.06.020 (2012).

9 Muzika, F., Bansagi, T., Jr., Schreiber, I., Schreiberova, L. & Taylor, A. F. A bistable switch in pH in urease-loaded alginate beads. *Chem Commun (Camb)* **50**, 11107-11109, doi:10.1039/c4cc03936j (2014).

10 Schiesser, W. E. *The numerical method of lines: integration of partial differential equations*. (Academic Press, 1991).
